# Evaluating eye health care services progress towards VISION 2020 goals in Gurage Zone, Ethiopia

**DOI:** 10.1186/s12913-022-08144-6

**Published:** 2022-06-10

**Authors:** Jibat Gemida Soboka, Tiliksew T. Teshome, Omar Salamanca, Alana Calise

**Affiliations:** 1 Department of Ophthalmology, College of Health and Medical Science, Haramaya University, Harar, Ethiopia; 2 Department of Ophthalmology, School of Medicine, College of Health Sciences, Addis Ababa University, Addis Ababa, Ethiopia; 3 Orbis International, New York, NY USA; 4 Service of OphthalmologyGrupo de Investigación en Visión Y Salud Ocular, VISOC, Universidad del Valle, Cali, Colombia

**Keywords:** Eye health care services, Progress evaluation, Gurage Zone, Vision 2020

## Abstract

**Background:**

Ethiopia signed the VISION 2020 Global Declaration and launched its eye health program in 2002. Since then, there has been limited systematic and comprehensive evaluation of the progress towards VISION 2020 goals in Ethiopia.

**Objective:**

To evaluate Gurage Zone progress towards VISION 2020 targets and process indicators.

**Method:**

An institutional-based cross-sectional study was conducted among all public and private eye health care facilities in the Gurage Zone within the Southern Nations, Nationalities, and People Region of Ethiopia. The evaluation protocol was adopted from the VISION 2020 situational analysis data collection tool. We used this structure to evaluate progress in terms of human resources for eye health, infrastructure, and service delivery at the zonal health office and health facilities. At the time of the study, Gurage Zone had a 1.7 million catchment area population. There were a total of five eye care centers, one of which was established by a non-governmental organization. Three of these facilities were secondary eye care centers with an operating theatre and two facilities were primary eye care centers. At the zonal level, there was no survey data available on the prevalence of blindness.

**Result:**

There was no systemic evaluation of VISION 2020 process indicators. The budget allocation specific to eye health care was less than 0.7% of the total budget of the zonal health office. The human resources for eye health (HReH) in the catchment area were: one ophthalmologist, two cataract surgeons, five optometrists, and 12 ophthalmic nurses, which is below the VISION 2020 targets for HReH. In terms of equipment, neither primary eye care center had a slit lamp biomicroscope, and two of the three secondary eye care centers did not have intraocular pressure measuring equipment. Only one secondary eye care center was providing glaucoma surgical services, and no center provided emergency and elective pediatric surgery. The cataract surgical rate determined by the study was 1967.

**Conclusion:**

Gurage Zone showed significant improvement in terms of cataract surgical rate. But it had not achieved VISION 2020 goals in terms of critical HReH and service delivery. We recommend that the zonal health office carries out a focused and baseline evaluation of eye health care service achievements.

**Supplementary Information:**

The online version contains supplementary material available at 10.1186/s12913-022-08144-6.

## Introduction

The World Health Organization (WHO) and the International Agency for the Prevention of Blindness (IAPB) launched “VISION 2020: The Right to Sight” in 1999. The objectives were to eliminate avoidable blindness by 2020 and prevent a projected doubling of the burden of visual impairment between 1990 and 2020. The major causes of blindness targeted were cataract, trachoma, onchocerciasis, childhood blindness, and refractive error [1].

Because of population growth and aging, the number of blind and visually impaired people is increasing at an alarming rate. These observations highlight the importance of responding to the WHO Global Action Plan 2006–2011, by scaling up current global, regional, and national efforts to eliminate the burden of avoidable blindness and vision impairment [2].

Eye health promotion, prevention, treatment, and rehabilitation are all part of a comprehensive eye care service package. The integration of these services into national health systems is a critical prerequisite for achieving the VISION 2020 goals. All 193 WHO member states formally committed to investing in eye care, and many countries established VISION 2020 committees and developed national eye care plans. However, implementation of these plans varied greatly across countries and remains the most difficult challenge to achieving the set goals [1].

There are still large populations in Sub-Saharan Africa who have limited or no access to eye care. While progress has been made with respect to eye care access in Ethiopia, there are lessons from these accomplishments that will help Ethiopia get closer to achieving the VISION 2020 goals. The VISION 2020 strategy specified measurable objectives such as impact indicators (for example, the number of cataract surgeries completed) and process indicators (for example, human resource development, or the number of ophthalmologists per million people) [1, 4, 5]. In 2002, the Ethiopian government signed the VISION 2020 Global Declaration and launched its eye health program. The national strategic plan for blindness prevention prioritizes three key areas of activity: controlling diseases that cause avoidable blindness; developing and deploying human resources for eye health (HReH) delivery; and developing and strengthening infrastructure and appropriate technology for eye health [6].

In this study, we evaluated eye health and progress towards VISION 2020 goals in terms of HReH, infrastructure, and service delivery in the Gurage Zone of the Southern Nations, Nationalities and People Region of Ethiopia (SNNPR).

## Methods

### Aim of study

The aim of the study was to evaluate Gurage Zone progress towards VISION 2020 targets and process indicators.

### Study design

We conducted a facility-based cross-sectional study using quantitative and qualitative methods in all eye care (ophthalmic) centers in the Gurage Zone as of May 2018.

### Study area

**Fig. 1:**
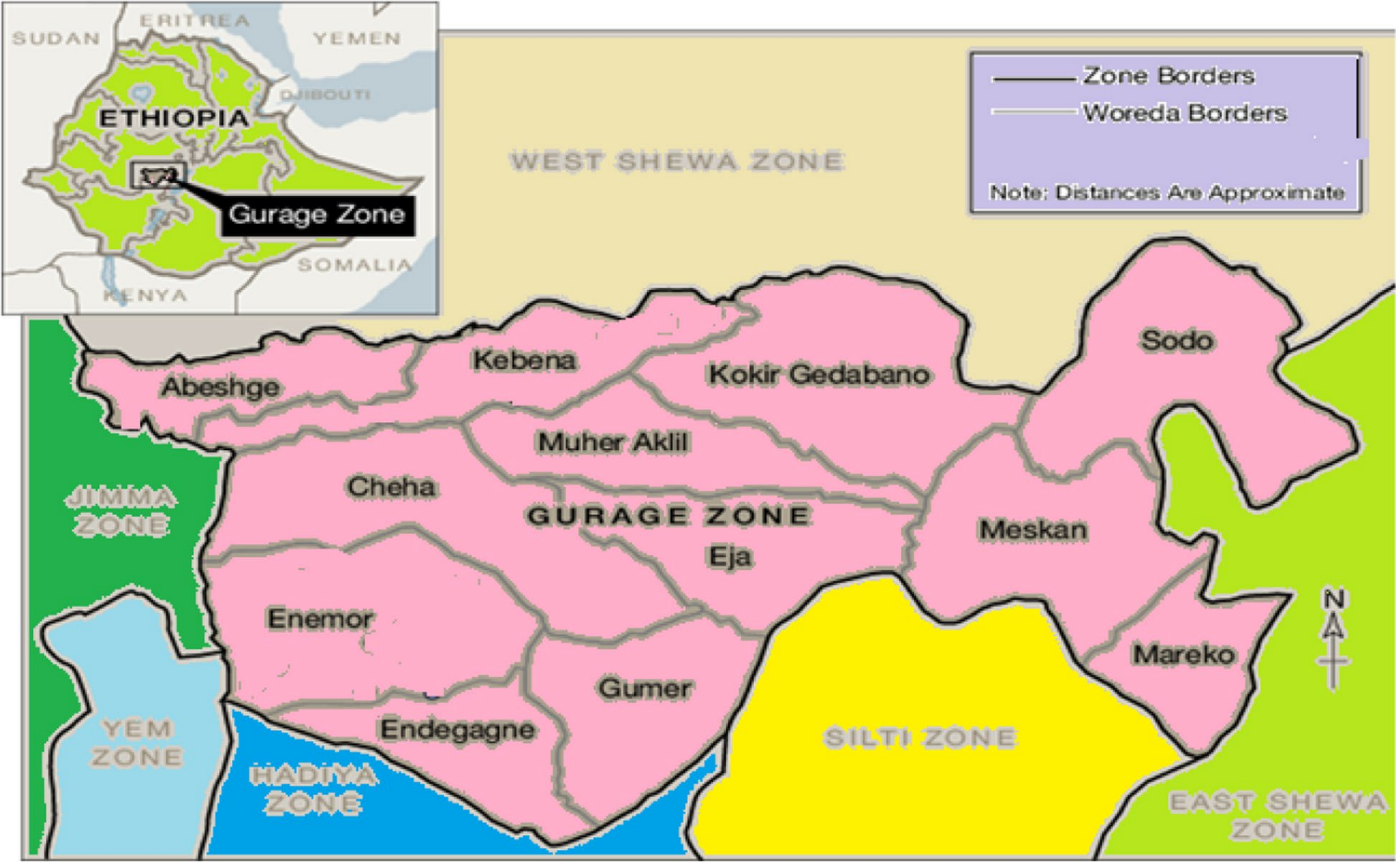
Map of the Gurage Zone, SNNPR, Ethiopia

The Gurage Zone is located 158 km south-west of Addis Ababa in the SNNPR in Ethiopia and has a total area of 5932 square kilometres (Fig. 1). According to projections from the 2007 population census by the Central Statistics Agency (CSA), the total population is 1,713,076, of which 839,406 (49%) are males and 873,670 (51%) are females [7].

A list of government and non-governmental organizations (NGO) health care facilities providing ophthalmic services was obtained from the Gurage Zone health office. Upon visiting the facilities, contact persons were identified by the principal investigator.

### Data collection

A questionnaire was administered during face-to-face interviews with the eye clinic head. The questionnaire was adopted from the VISION 2020 situation analysis data collection tool and was divided into four sections, including general information, human resources, equipment, and services [8, 9].

General information about the facilities included location, main sources of funding, catchment area population, number of beds, availability of outreach services, and number of patients seen per year. For equipment, a list of items considered essential for a functional eye unit was derived based on the guidance by the International Agency for the Prevention of Blindness (IAPB) [10]. The objective was to assess how many pieces of equipment were available and whether they were considered to be in optimal working condition. Information on the types of eye health care services offered was collected, including refraction and spectacles dispensing, population screening, low vision services, and surgery for cataract, trichiasis, and glaucoma. In the last section of the questionnaire, participants were asked to list the number of human resources for eye health available by role, including information on nationality, gender, place of training, and years of practice.

Eye care service management in the Gurage Zone was evaluated using parameters of condition management, stakeholder relationships, policy and regulation, and financing.

### Inclusion and exclusion criteria

Ophthalmic centers in Gurage Zone which were operational during the study period were included in the study. Health care facilities without trained eye care professionals and ophthalmic centers which were not operational during the study period were excluded.

Targets and process indicators based on VISION 2020 goals were calculated for the Gurage Zone based on WHO expert group recommendations for Sub-Saharan Africa established in 1997. The practitioner per population ratio was calculated and compared with the respective VISION 2020 targets [3].

### Data analysis

Data entry and analysis was done using SPSS version 21. After cleaning the data, frequencies and percentages were calculated to all variables which were related to the objectives of the study, and presented using Summary tables, graphs and charts for descriptive purpose.

### Operational definitions

Eye health care services are health care services related to the examination, diagnosis, treatment, and management of conditions, illnesses, and diseases of the eye and related structures.

Primary eye care is prevention and non-surgical treatment of the most common eye conditions and referral for surgical and advanced treatments.

Secondary eye care includes primary eye care services and some surgical services for the most common eye conditions, such as cataract and glaucoma.

Tertiary eye care comprises all subspecialty eye care services, including advanced diagnostic, medical, and surgical treatment of most eye conditions for both children and adults.

## Result

### General information

In 2018, Gurage Zone had a total of 71 health facilities, of which 64 (90.1%) were public and seven (9.9%) were NGOs. Five hospitals were primary care hospitals, and one was a secondary care hospital. Five (7%) facilities provided eye care services.

There has been no survey conducted on the prevalence and causes of blindness specifically by the Gurage Zonal Health Bureau. Instead, there were zonal health office reports indicating that cataract and trachoma were the top causes of blinding disease. A focal person was assigned to the zonal office in the neglected tropical diseases division to focus on eye health and prevention of blindness. The focal person had awareness of the VISION 2020 program. However, there was no VISION 2020 committee established at the zonal level and there were no prior systematic evaluations of eye care services conducted according to VISION 2020 objectives in Gurage Zone.

Orbis International and Christian Blind Mission (CBM), international non-profits, supported a trachoma prevention and treatment program as key partners of the zone health bureau. Partner support included finance, training, and program evaluation. The partners made progress and played a crucial role in combating trachoma in the Gurage Zone. There was no comprehensive eye care service regulation program based on written protocol and code of conduct [11]. Despite the absence of a standardized comprehensive eye care service system, the zone conducts an evaluation of the prevalence and management of active trachoma and an assessment of the availability of Trachomatous Trichiasis (TT) surgical coverage every three years.

The eye care services budget of the zonal office in 2018 was 0.69% of the annual capital budget. The budget allocation was primarily for the TT program. There was no budget allocation for medical equipment and technology transfer. The budget support from partners was one hundred times higher than the government’s budget allocation. The strongest secondary eye care unit (SECU) in the zone was the one that had an ophthalmologist and was supported by a partner with finance, equipment procurement, and management. Despite regular trachoma activities and cataract monitoring, there were no eye health promotion activities and no national eye care quality standards to follow.

#### Eye care centers

Of the five eye care centers, there were two were primary eye care centers, three were secondary eye care centers, and zero tertiary eye care centers. The five primary and secondary facilities saw 118,900 patients in the 2017/2018 budget year. On average, the facilities had been open for 11.4 years. The first eye care center in the zone was established in 1995 and the rest were established after the launch of VISION 2020. All the eye care centers were supported by government and NGO funding and planning. The government provided 80% of the funding, with NGOs providing the remaining 20%. Orbis International, CBM, and Light for the World were the NGOs supporting the centers. One secondary eye care center had 60 inpatient beds available. The inpatient bed to population ratio was 1:28,550. Outreach services were provided by all but one of the eye care centers, and those services included cataract surgery, trachomatous trichiasis (TT) surgery, and refraction.

**Fig. 2:**
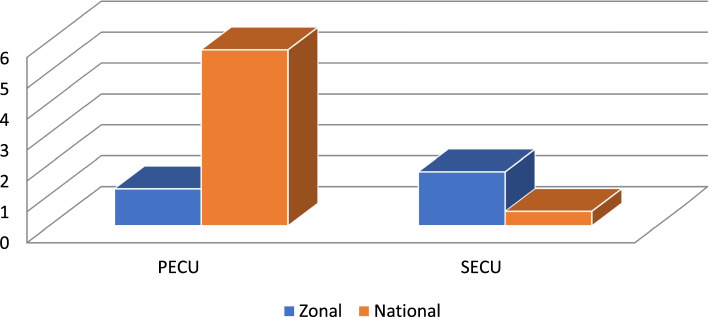
Number of Eye Care Centers, comparison between National and Gurage Zone per million population, Gurage Zone Ethiopia 2018

There were only two primary eye care units (PECUs) for a catchment area of more than 1.7 million people. This was by far below the recommended number of primary eye care centers for this catchment area. The numbers of PECU and SECU per million populations at the zonal level were 1.2 and 1.75, respectively. The overall fulfilment of PECU and SECU as per the national standard which was adopted from VISION 2020 in terms of human resources, services, and equipment was 42.8% and 55.5%, respectively (Fig. 2).

### Human resource

Of the 3,700 health care providers in the Gurage Zone, 23 were eye care workers (0.62%), of whom 16 (69.5%) were males and 7 (30.5%) were females, giving a male to female ratio of 2.3:1. There was one ophthalmologist (0.15:250,000) who was working in an NGO hospital, three cataract surgeons (0.4:250,000) (one working in an NGO hospital), 5 optometrists (0.17:50,000) (three working in an NGO hospital), and allied eye health professionals (0.7:100,000) (one ophthalmic clinical officer and five ophthalmic nurses working in an NGO hospital). There was no specific eye health human resources strategic plan prepared for or implemented at the zonal level. There has been an increasing trend in the number of TT surgeons over the past five years. There were a total of three ophthalmic nurses one ophthalmologist who were practicing in primary eye care centers. There were three cataract surgeons, five optometrists, and nine ophthalmic nurses practicing in secondary eye care centers.

All eye health care workers were Ethiopians who were trained in Ethiopia. The places of training were: Gondar University, Quiha Hospital, Addis Ababa University, Jimma University, Alert Hospital, Yirgalem Hospital, and Hawassa University. One maintenance technician was trained in India. On average, the healthcare workers had 10.4 years of experience, with a range of one to 23 years of practice. The monthly salary of the healthcare workers ranged from $160 USD to $1805 USD.

The available human resource for eye care at zonal level was below the target of the VISION 2020 process indicators across all professions. The number of ophthalmologists and optometrists was critically low. The ratios for ophthalmologists and optometrists were 0.15:250,000 and 0.35:100,000, respectively (Table 1).

**Table 1:** Comparison of eye care personnel working in Gurage Zone and the VISION 2020 recommendation—May 2018

Profession	Number of Practitioners	VISION 2020 Target Number of Practitioners	Deficit (%)	Need Met/Unmet
Ophthalmologist	1	7	85.7	Unmet
Cataract surgeon	3	7	57	Unmet
All surgeons	4	7	42.8	Unmet
Ophthalmic clinical officers (OCO) and nurses (ON)	12	17	29	Unmet
Optometrist	5	17	70.6	Unmet
Manager	1	2	50	Unmet
Technician	1	3	66.7	Unmet

**Fig. 3:**
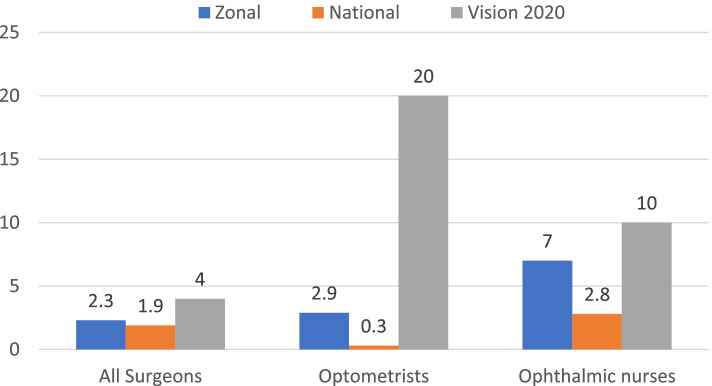
Comparison of human resources per million populations at the zonal level, national, level and VISION 2020 targets

The number of professionals per million population at the national level was comparable to Gurage Zone, and still below VISION 2020 targets (Fig. 3).

### Equipment

PECUs had 20% and SECUs had 70.4% of the necessary equipment, as per minimum national standards. Of all the available equipment in the facilities, 82% was functional, 9.8% needed repair, and 8.2% was beyond repair Neither primary eye care center had a slit lamp biomicroscope. Only one SECU had intraocular pressure (IOP) measurement equipment, the PECUs and other SECUs did not have IOP devices. Only one center had an equipment maintenance technician (Table 2).

**Table 2:** Equipment quantities at eye care centers in Gurage Zone Ethiopia, 2018

Equipment	Total Eye Care Centers (*n* = 5)	
	Equipment Quantity (n)	Eye Care Centers (n%)
Loupe	6	3 (60%)
SLM	7	3(60%)
Applanation Tonometer	3	1(20%)
Direct Ophthalmoscope	14	5(100%)
Indirect Ophthalmoscope	1	1(20%)
Trial Lens Set	10	4(80%)
Trial Frame	7	2(40%)
Cross Cylinder	5	2(40%)
A Scan	4	3(60%)
B Scan	0	0
Operating Microscope	6	3(60%)
YAG Laser	0	0
Cataract set	29	3(60%)

### Services

Sixty percent were providing school screening, refraction services and cataract surgery. All the SECUs had operating theatres. Only one SECU provided inpatient care, low vision, and glaucoma surgery services. TT surgery was provided in all eye care centers. No SECU provided elective and emergency pediatric surgery, vitreo-retinal surgery, pan retinal photocoagulation, YAG capsulotomy or exenteration services.

#### Cataract surgery

**Fig. 4:**
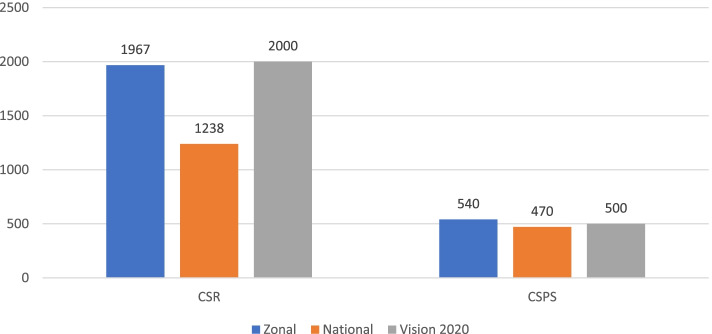
CSR and cataract surgery per surgeon by Gurage Zone data, National data, and VISION 2020 targets in Ethiopia, 2018

The total number of cataract surgeries performed in Gurage Zone in the previous calendar year was 3,373, including cataract surgery performed at outreach. From these, 2163 (64.2%) surgeries were done at a SECU and 1210 (35.8%) were done at an outreach event. The cataract surgical rate (CSR) was 1,967. There was no data to calculate cataract surgical coverage (CSC). The average patient waiting period for cataract surgery was 12 days. The intraocular lens (IOL) implantation rate was 95%. All the centers had quality control mechanisms for cataract surgery outcomes, including IOL implantation rate, outcome tally, and complication auditing system. The zonal CSR and cataract surgery per surgeon (CSPS) data was compared with the national level data and VISION 2020 targets (Fig. 4).

#### Refraction

In the Gurage Zone, only secondary eye care centers provide refraction services. Two of the three secondary eye care centers had optical workshops, but only one was functional. Pediatric refraction and cycloplegic (retinoscope) refraction were available only in one of the SECUs. All secondary eye care centers had services for eyeglass prescriptions in their compound, but only one had pediatric glass prescription services.

## Discussion

This is the first study to be conducted in Ethiopia at the zonal level with respect to VISION 2020 progress evaluation. We assessed progress toward VISION 2020 goals in terms of health system management, eye health care service delivery, eye health human resources, and infrastructure.

The zonal health bureau did not establish a VISION 2020 committee, which is one of the top priorities for managing and evaluating eye health. There was a national VISION 2020 committee but organizational cascading did not reach the zone. IAPB and WHO recommended the implementation of VISION 2020 and implementation should have been planned at the district level [12].

The budget allocation specific to the eye health services was 0.69% of the total annual capital zonal budget. International partners, particularly Orbis International and CBM, supported more than 72% of the annual zonal for eye care service improvement. The budget support from these partners helped the zonal health office especially in alleviating the burden of trachoma and trachoma related complications. The strongest SECU in the zone was supported by CBM, which was providing financing, equipment fulfilment, and administrative support. This showed that government priority for eye health was not sufficient.

The data from this study infers that NGO support is a central support system for eye health in Gurage zone. The goal would be to reduce reliance on NGO support and transfer roles to the government. Too much NGO support can limit the sustainability of healthcare programming, and it is desirable that health programming be maintained, funded, staffed, and led by government public health policies. NGO support can change unexpectedly due to external forces, and so building in sustainably funded and staffed eye care services into government budgets will ensure more consistent access to eye care services for the population.

Of all health care facilities in Gurage Zone, only 7% were providing eye care. This shows low integration of eye health care services within the existing health care system. This was a failure of one of the three main national strategic eye health plans drafted in 2006 [6]. Overall, in terms of available human resources and equipment fulfilment at the facility level, SECUs and PECUs achieved 55.5% and 42.8%, respectively.

In Gurage Zone, there are no tertiary eye care centers available. The lack of tertiary care facilities in general may be due to the population size, geographical dispersion of the population, and/or budget constraints. Without a tertiary care facility, it is difficult to provide specialized eye care to the population in this area. Those with eye health pathologies that require tertiary care currently need to travel to receive care or go without. This impacts many areas of an eye patient’s life and future. Building up tertiary care capacity for eye care in Gurage zone, as well as other areas in Ethiopia, is essential for comprehensive patient care and quality of life for the population. In our study we found that eye health care integration to existing health facilities was very low. Integration of eye health care through expansion of PECU and SECU in the existing health facilities, is the viable option to improve eye health care services in the zone.

The HReH in Gurage Zone was critically low. The number of available eye health professionals were below the target of VISION 2020 in all categories. There was only one ophthalmologist (0.15:250,000) in the zone. This HReH finding is consistent with data from many Sub-Saharan African countries, according to a study conducted by Palmer et al. [13]. The number of optometrists was also very low, at 0.17:50,000 population. In the study, Palmer et al. concluded that most sub-Saharan countries will not achieve the VISION 2020 targets by 2020. Ethiopia is currently half-way to meeting VISION 2020 target. At the current eye care practitioner growth rate, the VISION 2020 target for human resources in eye health will be met by 2034 [13, 14].

There has been a positive trend in the number of trained human resources for eye health in Ethiopia [16]. Many eye care teams are located in major cities, but it is expected that the increasing number of trained individuals will have a cascading affect to other geographical areas in Ethiopia. Primary, secondary, and remote tertiary facilities must carefully weigh the offers and incentives that they provide to existing and upcoming eye health personnel to attract and retain trained and specialized staff.

Equipment fulfilment in both PECUs and SECUs as per national standards for eye care services was 20% and 70.44%, respectively. It is of concern that PECUs did not reach 50% of the national standards in terms of infrastructure, integration, and human recourses. The SECUs were not consistently providing elective pediatric and vitreo-retinal surgery due to the lack of necessary equipment and trained personnel.

There were a total of 3,373 cataract surgeries performed in 2017. Sixty-four percent of surgeries took place with standard SECU operations, while 35.8% were completed during outreach campaigns. The CSR was 1967, which corresponded to the Vision 2020 target of 2000 and showed significant improvement from CSR before 20 years which was 303 [15]. The number of surgeries performed on an outreach basis was high. This might affect the progress in achieving consistently sufficient CSR in the future. Refraction services were provided in all SECUs, but no PECUs provide refraction services, which limits patient access. This finding was contrary to the expected national standard, in which refraction services are a primary activity at PECUs.

Eye health is multi-faceted, and while this paper documents the progress in Gurage zone compared to the VISION 2020 framework and targets, there are other frameworks and concepts to consider when measuring progress with respect to eye health. The data presented here is a baseline to monitor the progress of eye health trends in Gurage zone, and indirectly serve to contribute to the understanding of eye health in Gurage zone.

## Conclusion

The Gurage Zone showed a significant improvement in terms of cataract surgical rate within the past 20 years. However, the number of HReH, at primary and secondary eye care units was below the VISION 2020 targets. Our study showed that the Gurage Zone has some areas in need of improvement in order to achieve the VISION 2020 targets in terms of human resources, infrastructure, and integration of eye care services to existing health facilities.

## Recommendation

It is recommended that the Gurage Zonal Health Office conduct a focused and baseline evaluation of eye health care service achievements. The zone should expand the number of eye care centers, especially primary eye care units, and HReH. We also recommend that the Ministry of Health conduct a national eye care service evaluation.

## Strength and limitation

The strength of the study is its timeliness and accurate description of current situation. The major limitation is lack of baseline studies with respect to VISION 2020 indicators and goals prior to implementation of VISION 2020 programs. This study has limitations, because despite being conducted prospectively, some data were based on secondary data sources, which may not have all the updated information or that required to estimate some parameters. There was no verification of the information collected in the questionnaire, so it may have recall biases. On the other hand, the recommendations for VISION 2020 Goals are standard and may not reflect the local realities/needs of each of the regions and it is possible that those complexities are not fully contemplated in this report. The effect of the outreach campaigns for cataract surgery modifies the CSR, but the frequency of these activities for the Gurage zone and the total impact on the eye health indicators are unknown.

## Supplementary Information

**Additional file 1.** 

## Data Availability

The datasets used and/or analysed during the current study available from the corresponding author on reasonable request.
