# Supplementary material for: Evaluating eye health care services progress towards VISION 2020 goals in Gurage Zone, Ethiopia
Source: BMC Health Serv Res. 2022 Jun 10;22:768. doi: 10.1186/s12913-022-08144-6 (PMC9188140; doi:10.1186/s12913-022-08144-6)
Supplement: Supplementary file 1 — Additional file 1. [file 12913_2022_8144_MOESM1_ESM.docx]

**Annex A**

**Vision 2020 Indicators**

Supplementary Table 1: Eye Health Work Force/Human Resource for Eye Health/recommended in Gurage Zone Ethiopia

| **Eye Health Cadre** | **Key Functions** | | **Targets** | **Current National HReH profile** | | |
| --- | --- | --- | --- | --- | --- | --- |
|  |  |  |  | **Recommended** | **Available** | **Gap** |
| Ophthalmologists | Leadership, Surgery | | 1/250,000 | 7 |  |  |
| Optometrists | correction of RE | | 1/250,000 | 7 |  |  |
| Cataract surgeons | Leadership, Surgery | | 1/250,000 | Complementary  of  Ophthalmologists |  |  |
| Allied Eye Health professionals (Ophthalmic nurses, officers) | Diagnosis, treatment | | 1/100,000 | 17 |  |  |
| Primary Health Care Workers (IECWs) | Basic treatment and referral | | 1/10,000 | 171 |  |  |
| Community Health workers (HEWs) | Basic treatment and referral | | 1/1000 | 1710 |  |  |
| In patient bed | Treatment | | 1/20,000 | 85 |  |  |
| Ophthalmic OR | Treatment | | 1/1,000,000 | 2 |  |  |
| Optical workshop | | Diagnostic, treatment | 1/1,000,000 | 2 |  |  |
| CSR | | Treatment | 2000 | 3420 |  |  |

Fifty percent of secondary eye care facilities should have a full time manager and equipment technician

**Annex: B**

**Zonal Health Office Questionnaires**

1. Disease

Is there survey conducted on Prevalence of blindness in the zone?

Yes No

If yes, mention the survey data

What is the Proportion of blindness due to cataract?

Top causes of eye morbidity and blinding diseases?

1. Health system profile and background characteristics

- Total health facilities in the zone
- Number of health facilities providing eye care

1. Stakeholder relationship and support

- Which partners are supporting the zone?
- Mention the type of support?

1. Is their periodic Regulation of the eye care?

Yes No

- If yes, check for the following
- Protocol
- Standards
- Codes of conduct
- Certification

1. Eye health financing

- Total health expenditure ( annual zonal health office budget)
- Does the budget of the zonal health office include a specific allocation for eye care services?
  - Yes No
  - If yes, what percentage of the budget?
- Total expenditure pharmaceutical, medical products and technologies specifically for eye care?

1. Policy

- Do you know vision 2020?
- Is there a zonal plan for eye health and prevention of blindness?
  - Yes No
  - If yes, state who implement and monitor the plan
- Is there focal person for eye health and prevention of blindness at zonal health office?
- Is there a formal referral pathway?
- Is there eye health promoting activities?
- Existence of national eye care quality standard, adapted to local level?

1. Human resource
   - Does health human resource strategic plan exist?

- Total no of eye health care providers?
- Trend in eye care workers over the past 5 years
  - Compare with WHO/IAPB stand
  - Profession
  - Primary, secondary and tertiary service
  - Area(province)
  - Urban/rural
  - Private/ public

1. Data sources

- Availability and accessibility of data sources specifically for eye are?
- Percentage of fascilities reporting eye care information (CSR,TT….)

1. Provision of eye care in zone at the three levels of care

Supplementary Table 2: Provision of eye care in zone at the three levels of care

| Eye care | 1^0^ care | 2^0^ care | 3^0^care |
| --- | --- | --- | --- |
| Government |  |  |  |
| NGO |  |  |  |

Annex: C

**Facility Questionnaire**

1. **General Information**

Supplementary Table 3: General information about health facilities, Gurage Zone

| Name of Facility | Address | City |
| --- | --- | --- |
| Region | Name of contact person | Email |
| Province |  |  |
| Established Yr |  |  |

| Support/Affiliation | Main source of Funding |
| --- | --- |
| Government | 1. |
| Non Gov. | 2. |
| Private | 3. |

| Facility Type | For Hospital/Clinic |
| --- | --- |
| Hospital | Level of care Primary Secondary Tertiary |
| Health Center | Inpatient Care Yes No |
| Clinic | No.Beds |
| Optic center | Outreach Services Yes No |
|  |  |

| Patient Population | | | |
| --- | --- | --- | --- |
| Catchment area | Estimated number of patients per year: | Facility | Outreach |

1. **Equipment**

Indicate availability of the following:

Supplementary Table 4: Status of Ophthalmic Medical Equipment at health facilities, Gurage Zone

| Item | Functional Status | | | | | Comment |
| --- | --- | --- | --- | --- | --- | --- |
|  | Total | Excellent | Good | Need repair | Beyond repair |  |
| Loupe |  |  |  |  |  |  |
| SLM |  |  |  |  |  |  |
| Tonometer – Applanation |  |  |  |  |  |  |
| Tonometer – Schiontz |  |  |  |  |  |  |
| Ophthalmoscope – Direct |  |  |  |  |  |  |
| Ophthalmoscope – Indirect |  |  |  |  |  |  |
| Trial lens set |  |  |  |  |  |  |
| Trial frame |  |  |  |  |  |  |
| Cross cylinder |  |  |  |  |  |  |
| A Scan U/S |  |  |  |  |  |  |
| B Scan U/S |  |  |  |  |  |  |
| AB Scan U/S |  |  |  |  |  |  |
| Operating microscope |  |  |  |  |  |  |
| Cataract sets |  |  |  |  |  |  |

1. **Services**

Indicate the availability of the following

Supplementary Table 5: Eye care Services provided at health facilities, Gurage Zone

| Services | Availability | | Comment |
| --- | --- | --- | --- |
|  | Yes | No |  |
| Screening (e.g. school, community) |  |  |  |
| Refractive |  |  |  |
| Low vision |  |  |  |
| Specs dispensing |  |  |  |
| Cataract surgery |  |  |  |
| Trichiasis surgery |  |  |  |
| Glaucoma surgery |  |  |  |
| VR surgery |  |  |  |
| Laser PRP |  |  |  |
| Exentration/enuclation/evisceration |  |  |  |
| Other activities |  |  |  |

**Cataract**

Supplementary Table 6: Total number of cataract surgery performed in the past calendar year, Gurage zone

|  |  |
| --- | --- |
| Cataract Surgical Rate |  |
| ⃰Cataract Surgical Coverage |  |
| Waiting list time (average period to receive contract surgery in days) |  |
| IOL implantation rate |  |

Is the quality of cataract surgery services monitored?

Yes No

If yes How?

**Refraction**

Is there Optical workshop in the zone?

Yes No

If yes, How much?

Supplementary Table 7: Refraction services availability, Gurage Zone

| **Refraction services** | **Comment** |
| --- | --- |
| Is refraction service available? |  |
| Is pediatrics refraction service available? |  |
| Is cyclopentolate retinoscopic refraction available? |  |
| Is glass prescription available in the compound? |  |

1. **Human Resource**

- Total number of eye health care providers

Supplementary Table 8: Number and professional types of eye health care providers, Gurage Zone

| Role description* | Nationality | Gender | Place of training | Years of practice | Salary payer (eg; Gov, Donors, |
| --- | --- | --- | --- | --- | --- |
|  |  |  |  |  |  |
|  |  |  |  |  |  |
|  |  |  |  |  |  |
|  |  |  |  |  |  |
|  |  |  |  |  |  |
|  |  |  |  |  |  |
